# Standardizing fatigue-resistance testing during electrical stimulation of paralysed human quadriceps muscles, a practical approach

**DOI:** 10.1186/s12984-021-00805-7

**Published:** 2021-01-21

**Authors:** Martin Schmoll, Ronan Le Guillou, David Lobato Borges, Charles Fattal, Emerson Fachin-Martins, Christine Azevedo Coste

**Affiliations:** 1 INRIA – LIRMM, Université de Montpellier, Montpellier, France; 2 NTAAI, Faculdade de Ceilândia, Universidade de Brasília, Brasília, Brazil; 3 CRF La Châtaigneraie, Menucourt, France

**Keywords:** Fatigue testing, Functional electrical stimulation, Distributed stimulation, Spinal cord injury, Knee dynamometer, Dynamic movement

## Abstract

**Background:**

Rapid onset of muscular fatigue is still one of the main issues of functional electrical stimulation (FES). A promising technique, known as distributed stimulation, aims to activate sub-units of a muscle at a lower stimulation frequency to increase fatigue-resistance. Besides a general agreement on the beneficial effects, the great heterogeneity of evaluation techniques, raises the demand for a standardized method to better reflect the requirements of a practical application.

**Methods:**

This study investigated the fatigue-development of 6 paralysed quadriceps muscles over the course of 180 dynamic contractions, evaluating different electrode-configurations (conventional and distributed stimulation). For a standardized comparison, fatigue-testing was performed at 40% of the peak-torque during a maximal evoked contraction (MEC). Further, we assessed the isometric torque for each electrode-configuration at different knee-extension-angles (70°–170°, 10° steps).

**Results:**

Our results showed no significant difference in the fatigue-index for any of the tested electrode-configurations, compared to conventional-stimulation. We conjecture that the positive effects of distributed stimulation become less pronounced at higher stimulation amplitudes. The isometric torque produced at different knee-extension angles was similar for most electrode-configurations. Maximal torque-production was found at 130°–140° knee-extension-angle, which correlates with the maximal knee-flexion-angles during running.

**Conclusion:**

In most practical applications, FES is intended to initiate dynamic movements. Therefore, it is crucial to assess fatigue-resistance by using dynamic contractions. Reporting the relationship between produced torque and knee-extension-angle can help to observe the stability of a chosen electrode-configuration for a targeted range-of-motion. Additionally, we suggest to perform fatigue testing at higher forces (e.g. 40% of the maximal evoked torque) in pre-trained subjects with SCI to better reflect the practical demands of FES-applications.

## Background

Functional electrical stimulation (FES) has become a well-established tool in clinical rehabilitation of individuals with spinal cord injury (SCI). While FES has been shown to be beneficial to counteract muscular loss [1], increase strength [2] and increase the overall well-being—it still reveals severe weaknesses in more practical applications such as FES assisted cycling [3]. Although electrical stimulation allows to elicit high forces, it is very difficult to maintain these force-levels for longer durations—a requirement for any functional task. Muscular fatigue can be considered to be one of the main problems of FES and remains an open challenge. During voluntary contractions motor-units are recruited according to the Henneman´s size principle [4] and randomly switched so that each individual motor-unit is active only for a short amount of time. FES-induced contractions are unable to replicate such a recruitment pattern—motor-neurons are rather activated based on an individual threshold defined by axon-size and distance to the electrode. With conventional stimulation methods, the same motor-units are activated by every pulse, which causes a rapid onset of muscular fatigue. One strategy to counteract fatigue, known as distributed stimulation, is to reduce the number of stimulation pulses acting on a particular motor unit. The idea is to distribute stimulating pulses over multiple channels to activate different sub-units of a muscle, which was extensively described in [5]. Due to mechanical coupling inside of the muscle, distributed stimulation is able to generate strong fused contractions normally only achieved at higher frequencies.

When focussing on FES-cycling, the quadriceps muscles are the main actors for generating the pedalling movement, due to their functional role as knee-extensors. In addition they have a high muscular mass and their nerves are easy to access via surface electrodes. Several studies have shown improved fatigue-resistance when using distributed multichannel stimulation compared to conventional single channel stimulation [6–14]. Nevertheless, a clear comparison between the studies is difficult as most studies present different electrode configurations, stimulation parameters and methods for quantifying muscular fatigue.

In earlier studies, fatigue-resistance was quantified during continuous isometric contraction [12, 13] by measuring the duration until the measured torque drops below a predefined threshold, i.e. 70% of the initial torque. Both studies were able to demonstrate significantly longer fatigue intervals in SCI subjects, when using distributed stimulation. The average increases were reported between 26.2% (range 5.9–81%) [12] and 153.18% (range 66.7–255.34%) [13]. Although these results showed statistically significant improvements, continuous isometric contractions are almost never used alone in a functional application. In 2015, Downey et al. presented a somewhat more practical approach for fatigue-testing, by using intermittent trains of stimulation (5 s ON, 5 s OFF) to elicit isometric contractions at different stimulation frequencies (32 Hz and 64 Hz) [8]. The authors compared a conventional 2 electrode configuration against a distributed electrode-configuration with 2 anodes and 4 cathodes. Each individual distributed cathode thus received a fourth of the stimulation frequency applied during conventional stimulation. Besides a fatigue time (determined for a threshold of 80% of the initial torque), they also assessed a fatigue index (FI) which describes fatigue resistance as the quotient between final torque (mean of last 3 contractions) divided by initial torque (first contraction). A fatigue index of 1 therefore means no fatigue. In their participants with SCI, they found significant improvements in the fatigue time (181 ± 107% increase) and the fatigue index (0.17 ± 0.13 higher FI) for distributed stimulation (8 Hz per electrode) against conventional stimulation (32 Hz). Even more pronounced effects (fatigue time: 640 ± 445% increase; fatigue index: 0.24 ± 0.11 higher FI) were reported at higher stimulation frequencies (distributed stimulation: 16 Hz per electrode vs. 64 Hz during conventional stimulation). Nevertheless, fatigue-testing still was performed under isometric conditions. Ultimately, more recent studies began to investigate fatigue during a dynamic movement task [6, 8–11], which increases the complexity of the measurement setup. Although all studies concluded improvements for the use of distributed stimulation, it is important to note that most results were obtained in able-bodied healthy subjects. Only Laubacher et al. reported results for the population with SCI [9]. They claimed increased fatigue resistance during distributed stimulation in 3 out of 4 subjects, which was mainly supported by higher values of mean power (average power of all contractions) and final power (average of last 20 contractions). Out of the 8 investigated legs in their study, 5 revealed a higher fatigue index using distributed stimulation.

Another aspect which complicates the comparison of results between different studies is the great variation in initial values used for fatigue testing. Although all studies describe their criteria for selecting their initial values, only Bergquist et al. [6] and Downey et al. [8] put their results in correlation with a measurement of maximal torque (maximum voluntary contraction of able bodied subjects, MVC). Studies in SCI-subjects do not report such a correlation, which raises the need for standardization. This would improve comparability between studies and allow for a more meaningful interpretation of the results in the context of practical applications.

Our study aimed to accumulate previous knowledge, to generate a standardized method for investigating FES-induced muscular fatigue in individuals with SCI, during a dynamic movement task. To allow for better comparison between tested muscles, fatigue-testing was performed at a torque of 40% of the torque produced during a maximal evoked contraction. This level was chosen to represent the midpoint of the range for aerobic muscular endurance during dynamic actions, which was reported as 30–50% of maximal muscular tension in healthy subjects [15]. As other studies before, we were interested in the influence of different electrode configurations on muscular fatigue. Additionally we also wanted to assess the generated torque for different knee-extension angles, as this reflects an important marker of the stability of a certain stimulation technique. We further hypothesised that more targeted electrode configurations (i.e. electrodes in close proximity to the related motor-point) might have a beneficial effect on the torque-generation over a wider range of the knee-extension-angle.

## Material and methods

### Subjects

Three male participants with SCI (42.4 ± 6.9 years, body mass index: 26.6 ± 2.9) were recruited for this study. They were all familiar with electrical stimulation and conducted regular FES-induced strengthening training of their quadriceps muscles for 14 months prior to the study. Each of them gave written consent to the procedures performed in this study. The protocol was approved by the Ethics and Research Committee of the Faculty of Ceilandia, University of Brasília, Brasília, Brazil (CAAE: 09303218.6.0000.8093, Ethical Approval number 3.632.981). The inclusion criteria were: (i) complete spinal cord injury AIS grade A, (ii) single neurological level between C6 and T12, (iii) time interval since SCI > 12 months (with a stable ASIA motor score > 6 months), (iv) PENN-Scale < 3, (v) unrestricted joint movement, (vi) body-mass index < 30 and (vii) T-Score greater than -2.5. Exclusion criteria were: (i) acute muscle disease, (ii) cardiovascular disease, (iii) medication known to have a negative effect on bone stability, (iv) epilepsy, (v) fractures of the lower limbs within the last 12 months, (vi) wearing a heart pacemaker or other implants which contraindicate the use of FES. Although FES is considered a rather safe technique and widely used for rehabilitation of patients with SCI, the risk of a potential bone fracture [16, 17] needs to be considered carefully when recruiting participants. Apart from the medical criteria, subjects were required to participate on a regular FES-based training regime of the quadriceps muscles for at least 4 months in order to minimize a potential training effect induced by the measurements. Detailed information about the participants can be found in Table 1 and Additional file 1: Table S1.

**Table 1:** Subject specific data

Subject	Age	Gender	Body Mass Index	AIS Grade	Level of Lesion	Time since injury	FES training since	T-Score Whole body	T-Score Femur	BMD Whole body	BMD femur	PENN Scale
	years	-	kg/m^2^	-	-	years	months	-	-	g/cm^2^	g/cm^2^	-
P1	48.3	male	24.0	A	T7	7.7	14	-0.7	-1.3	0.902	0.907	1
P2	44.0	male	29.7	A	T6	4.1	14	1.0	-0.7	0.961	0.997	1
P3	34.8	male	26.0	A	T1	13.1	14	0.5	-1.1	0.941	0.946	1

### Measurement setup

**Fig. 1:**
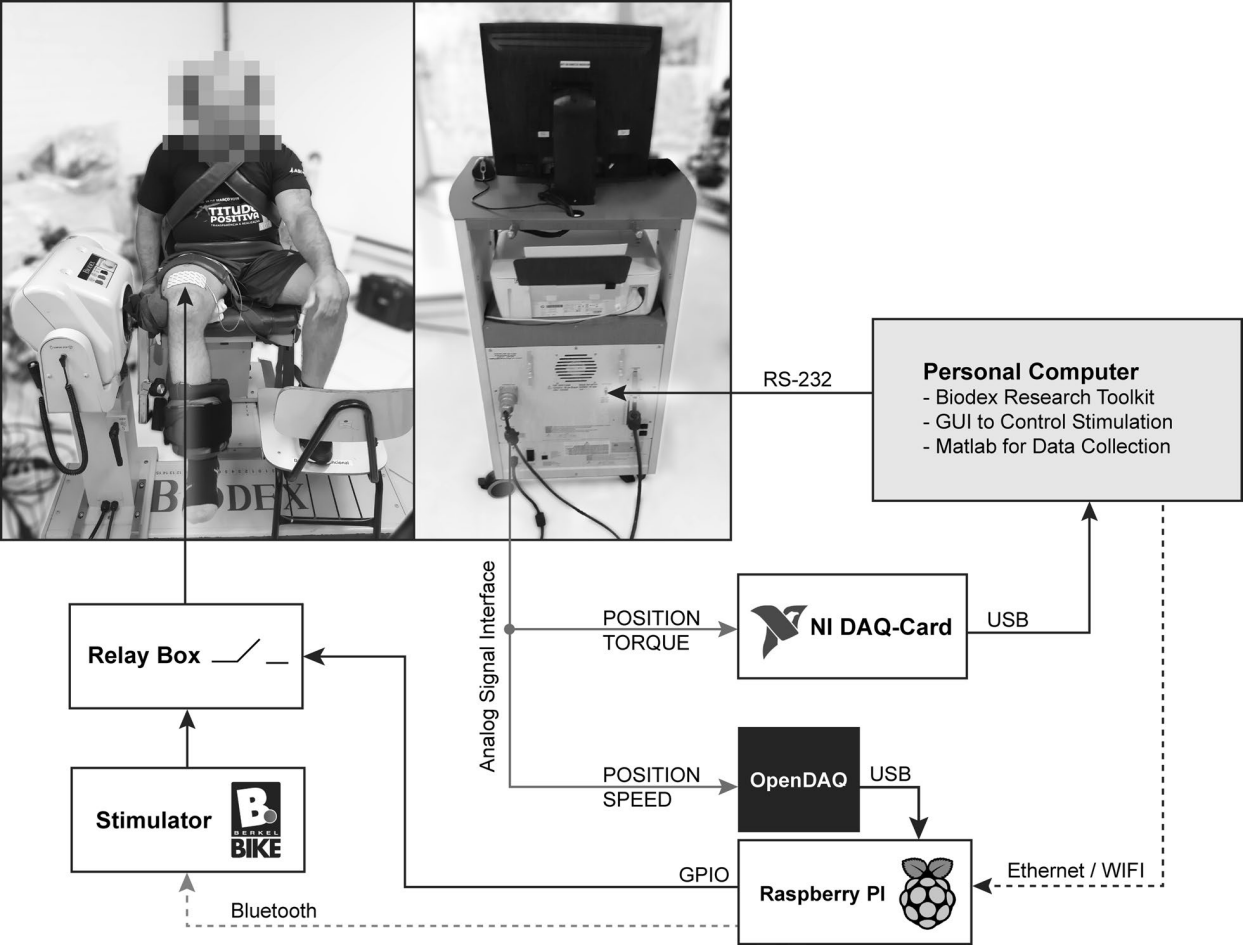
Measurement Setup. A standard personal computer was used to control the movement of the dynamometer while recording and displaying its data. The PC was further used to configure a raspberry PI responsible for delivering the stimulation. The raspberry processed signals from the dynamometer and controlled the stimulator and a relay-box to trigger stimulation at the desired positions

Measurements in this study were performed on an isokinetic dynamometer (System 4, Biodex Medical Systems, NY, United States). Participants were comfortably seated and additional cushioning was provided where needed. An overview of the measurement setup is presented in Fig. 1. The axis of the knee-joint was aligned with the rotational axis of the dynamometer. The measurement arm of the dynamometer was adjusted to be parallel to the frontal edge of the tibia. Using the company´s software (Research Toolkit v1.4, Biodex Medical Systems, NY, United States) on a standard personal computer (MSI GS60 2PE Ghost Pro, Micro-Star International, Zhonghe District, Taiwan) allowed to upload custom designed movement patterns to control the dynamometer via an RS-232 connection. The personal computer was the main point of user interaction responsible for movement control of the dynamometer, adjustment of stimulation parameters and data acquisition.

Each movement profile (sequence of consecutive positions) was generated with Matlab 2017b (MathWorks, Natick, Massachusetts, United States), stored in in a plain text file and uploaded to the dynamometer. For safety reasons the angular velocity was linearly increased or decreased within the first and last 100 ms of each movement to ensure smooth transitions. The current position, velocity and torque of the dynamometer were available via the “Analog-Signal-Access-Interface” from Biodex. A NI-DAQ card (USB 6218, National Instruments, TX, United States) controlled by a Matlab script, was used to record torque, position, stimulation current via a 100 Ω shunt resistor and a flag determining the stimulation period, at a sample rate of 2 kS / s. Besides recording, the Matlab script allowed to preview the data in real-time. After recording a full cycle (extension and flexion of the leg) a preliminary analysis was performed to automatically determine peak-torque for each contraction—a crucial feature for setting the stimulation amplitude during the experiments.

Position and velocity were additionally captured by a Raspberry Pi (Model 3B, Raspberry Pi Foundation, United Kingdom) using an USB data acquisition module (openDAQ, INGEN10 Ingenieria SL, Spain). The stimulation delivered to the participant was controlled by a Python program. The program communicated wirelessly via Bluetooth with the stimulator (FESBox 4, BerkelBike B.V., Netherlands) to update stimulation amplitude, phasewidth (PhW) and frequency (F). Further, it controlled a relay module (8 relay Module, AZ-Delivery Vertriebs GmbH, Deggendorf, Germany) which mechanically switched the stimulation to the electrodes. The Python program of the raspberry PI was controlled via a separate C# program running on the personal computer.

### Electrode setup and positioning

#### Detection of motor points

To allow for reproducible positioning of the electrodes, the motor points of the quadriceps muscles: vastus medialis, vastus lateralis and rectus femoris, were determined using a Quark stimulator (Model Dualpex 071, Quark Medical, Piracicaba, Brazil). The detection was conducted by the coordinator of the local facility who routinely performed this procedure on his patients. A 70 × 50 mm sponge electrode (stainless steel plate inside of a wetted sponge shell) placed ~ 50 mm proximal to the popliteal fossa served as anode. The stimulator´s cathode was connected to a custom-build motor point pen. Motor points were determined using monophasic, rectangular pulses (PhW = 10 ms, F = 4 Hz) at knee-extension-angles of 70° and 130°. Both points were marked and connected via a straight line, using the middle of this line as a reference point for the electrode placement. Motor points were determined and marked at the first session and remarked every day.

#### Electrode configurations

**Fig. 2:**
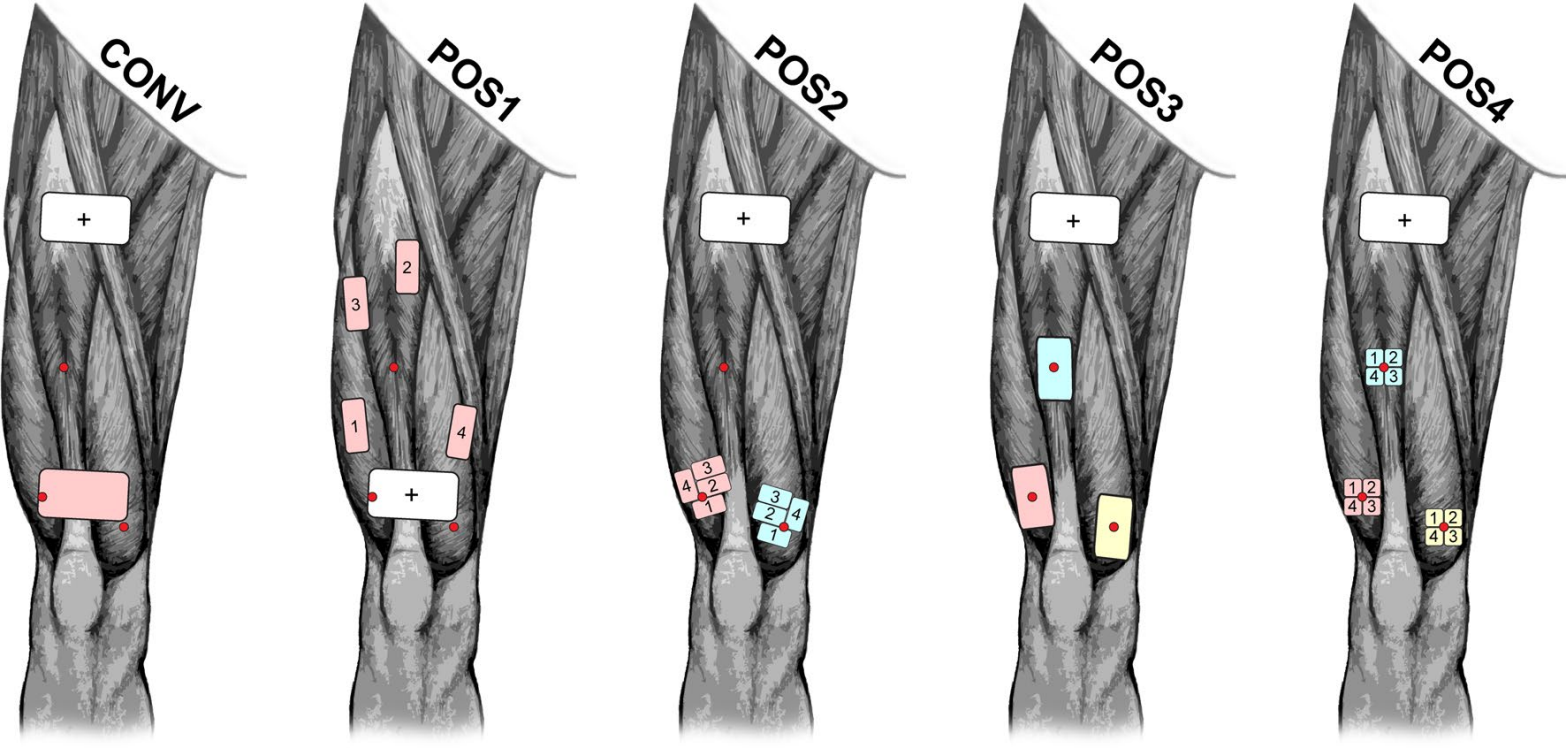
Electrode configurations. A graphical representation of the electrode placement for the individual electrode configurations. All configurations had a common anode (white “ + ” electrode) which served as reference electrode. Individual stimulation channels are marked by different colours. Numbers on the electrodes, indicate the sequential order of pulses delivered during distributed stimulation. The motor-points used for orientation of the electrode configuration are displayed with red-circles

The tested electrode configurations gather a selection of electrode placements partly based on literature and adapted to the current study (see Fig. 2). The self-adhesive hydrogel electrodes were supplied either by Axelgaard (Myotrode Platinum, Fallbrook, CA, USA) or Axion (Leonberg, Germany) and cut into the desired sizes. Besides the conventional electrode configuration (CONV), all electrode configurations correspond to a monopolar stimulation regime (i.e. big reference electrode, small active electrode). The reference electrode (68 × 125 mm) was always the anode (i.e. for a biphasic pulse, the anode receives the positive phase before the negative phase). Electrode-configurations which implemented distributed stimulation (i.e. POS1, POS2 and POS4) used an anti-fatigue-unit (AFU, Model: 3F-AFU-10, 3F-Fit Fabricando Faber, Belgrade, Serbia). The AFU distributed incoming stimulation pulses sequentially to 1 out of 4 output channels, i.e. each individual electrode within the distributed stimulation setup received a stimulation frequency of 10 Hz.

For the CONV electrode configuration two equally sized electrodes (68 × 125 mm, bipolar stimulation) were placed proximally and distally on the quadriceps muscle. CONV is characterised by an easy electrode placement and thus widely used.

Configuration POS1 was adapted from Malešević et al. [12] and Bergquist et al. [6]. Four small electrodes (33 × 75 mm) were placed according to the instructions of the authors. The electrodes were positioned on the proximal and distal part of the m. vastus lateralis, the proximal part of the m. rectus femoris and the proximal part of the m. vastus medialis. The widely spaced positioning of the stimulation electrodes in POS1 is intended to provide a clear separation of different pools of motor-units across different muscles of the quadriceps. Unfortunately we experienced technical issues with this electrode configuration which forced us to exclude it from the data-analysis (please find more details in the results and discussion).

Configuration POS2 was adapted from Laubacher et al. [9–11]. Four small electrodes (25 × 45 mm) formed a group and were aligned around the desired motor-point. Two of these groups were targeting the distal motor-point of m. vastus lateralis and the distal motor-point of m. vastus medialis. Distributed stimulation was delivered individually for each group (one channel and AFU per group). Both groups had a common reference electrode located on the proximal part of the quadriceps muscles. POS2 aims to recruit different motor-units within two different motor-points.

Configuration POS3 used 3 smaller (50 × 90 mm) electrodes to target the distal motor-point of m. vastus lateralis, the distal motor-point of m. rectus femoris and the distal motor-point of m. vastus lateralis. Each electrode was supplied by an individual stimulation channel while having a common reference electrode located on the proximal part of the quadriceps muscles. With POS3 we wanted to investigate if an electrode placement directly on the motor-point has an effect on fatigue resistance.

Configuration POS4 was targeting the same motor-points as POS3 but used distributed stimulation. Four small electrodes (25 × 25 mm) formed a group and were aligned around the desired motor-point. Distributed stimulation was delivered individually for each group (one channel and AFU per group). All groups had a common reference electrode located on the proximal part of the quadriceps muscles. POS4 was intended to extend the concept of distributed stimulation onto three different motor-points.

### Testing protocol

All measurements sessions have been performed with the system depicted in Fig. 1. The stimulator (Berkelbike FESBox) delivered symmetric, rectangular, biphasic pulses with a phasewidth of 400 µs. Each testing session consisted of two parts and was conducted for left and right leg. Both legs were tested separately during the same session. Between consecutive sessions a minimal rest of 24 h was ensured.

The first part was intended to prepare the subject (warm up) and to determine a baseline (maximal evoked contraction) individually for each session. The first part was always conducted with the CONV electrode configuration.

In the second part we measured the isometric torque for different knee-extension angles (i.e. 180° indicates a full knee-extension) and conducted a dynamic fatigue-test. For these measurements, the electrode configuration was changed according to a randomized testing schedule. In order to detect potential training effects, each subject was measured with the CONV electrode configuration on the first (CONV1) and last (CONV2) measurement day. Randomisation was therefore only applied to electrode positions POS1, POS2, POS3 and POS4.

#### Warm up

A warm-up program was conducted using the CONV electrode configuration. The leg was moved passively (angular velocity 110°/s as used in [9–11]) by the dynamometer between 70° and 130° knee-extension-angle for 180 times. Stimulation was applied at a frequency of 20 Hz during every second knee-extension from 75° to 125° knee-extension-angle. The stimulation intensity was set to elicit a clearly palpable contraction. The purpose of the warm-up was to mobilize and prepare the leg muscles for the following testing and to lower a potential spastic reaction to the stimulation or movement.

#### Maximal evoked contraction (MEC)

The torque during maximal evoked contractions was determined using the CONV electrode configuration. Three short (500 ms) isometric contractions at a knee-extension-angle of 90°, were elicited at 120 mA at a stimulation frequency of 40 Hz. Between the contractions a rest of 1 min was incorporated to allow for full recovery of the neuromuscular system. The average of the 3 consecutive peak-torque values was considered to be the maximal torque production capability of the muscle (MEC) for the concerned session. Slightly higher torque-values might be achieved with higher stimulation parameters (e.g. amplitude, phasewidth and frequency), but for safety reasons we decided on these parameters, as they are used commonly as maximum values during practical applications (e.g. FES-cycling).

#### Torque to knee-extension-angle relationship

The relationship between knee-extension-angle and produced torque was assessed for all electrode configurations. The knee-extension angle represents the inner angle between thigh and shank which is different from the knee-angle (or knee-flexion angle) used in clinical applications. Therefore, a full knee-extension is reached at 180°, while lower values indicate a flexion of the knee. For the measurements, short (500 ms, F = 40 Hz) isometric contractions were elicited for different knee-extension-angles, ranging from 70° to 170° (in steps of 10°, two contractions per knee-angle). The muscles were given 30 s of rest between the contractions. The stimulation intensity was adjusted to produce a peak-torque of approximately 40% of the MEC at the lowest knee-extension-angle (i.e. 70° knee-extension-angle).

#### Fatigue testing

Fatigue measurements were performed for all electrode configurations. The dynamometer was programmed to move the leg between 70° and 130° knee-extension-angle (angular velocity 110°/s as used in [9–11]) for 360 repetitions. Stimulation was applied at a frequency of 40 Hz (for each channel) every second knee-extension from 75° to 125° knee-extension-angle in order to obtain the actively produced torque (see data analysis). The stimulation amplitude was adjusted prior to the actual fatigue test, targeting an active peak-torque of 40% MEC, during the dynamic movement. After a short rest of 2 to 3 min the fatigue-test was started.

### Data analysis

The torque recorded with the dynamometer is the sum of an active and a passive component. While the active torque was generated by the muscular contraction itself, the passive component was mainly caused by the weight of the leg and the fixation arm. The component of interest is thus the active torque only generated by the muscles.

For all isometric measurements the active torque was obtained by subtracting the constant passive offset (50 ms average of torque signal before onset of stimulation burst).

Dynamic movements (fatigue testing) required the recording of two complete movement-cycles (one active cycle with stimulation and one passive cycle without stimulation) to calculate the active torque. The active torque was obtained by subtracting the passive cycle from the active cycle. Traces of the active torque along with their corresponding peak-values were plotted during the experiment for every pair of cycles, allowing for visual inspection of the recently recorded active torque data. In offline post-processing an average of all recorded passive torque traces was used to obtain a more reliable estimation of the passive torque. Some contractions were manually excluded from analysis due to assumed spastic activity in the passive torque cycle (shape of trace visually very different from the average passive torque trace). Remaining contractions were normalized to the MEC of the corresponding session to allow for comparison between different legs. For every contraction peak torque and average torque (during stimulation) were determined. These values were used to calculate fatigue indices (FI) which were based on the means of the first 10 (τ_init_) and last 10 (τ_final_) contractions of each session (see Eq. 1). The fatigue index represents a measure of fatigue resistance, i.e. FI = 1 means no fatigue, while FI = 0 corresponds to complete muscular exhaustion (no active torque).

1 $$FI= \frac{{\tau }_{final}}{{\tau }_{init}}$$

Each individual leg was considered as an independent sample. Due to the low sample size (n = 6) normal distribution could not be guaranteed for every data-set. Therefore the non-parametrical Friedman test with a selected significance level of α = 0.05 was used to assess statistical differences between the different electrode configurations.

## Results

Within a period of 11 days we performed 6 measurements sessions (duration of a session: ~ 2.5 h) in 3 subjects with complete SCI (separate testing of left and right leg in each session). It was possible to conduct all sessions without complications (i.e. no fractures, no skin burns, no articular dislocations, no post-experimental oedema or inflammation).

### Maximal evoked contraction

MEC was comparable between the different subjects and ranged from 30 to 40 Nm (see Additional file 1: Table S1 for individual MEC values of each leg). MEC was recorded at the beginning of every test session, thus accounting for daily variations in muscular strength. From the first session (CONV1) to the last session (CONV2) the MEC decreased from 36.2 ± 5.0 Nm to 33.6 ± 3.5 Nm, but there was no statistical difference between sessions (p = 0.458).

### Stability of electrode configurations

Besides POS1, all electrode configurations were able to elicit stable strong fused contractions. However, with POS1 it seemed crucial to apply the distributed stimulation in an ordered manner (i.e. first pulse to electrode 1, second pulse electrode 2, and so on…). Although we kept the correct order as described in (1, 17), our setup did not allow for a particular starting electrode to be chosen. Thus we observed mostly unfused contractions and only sometimes strong fused contractions. Due to these problems we were not able to obtain reliable tetanic contractions and decided to exclude POS1 from the analysis.

### Torque to knee-extension-angle relationship

**Fig. 3:**
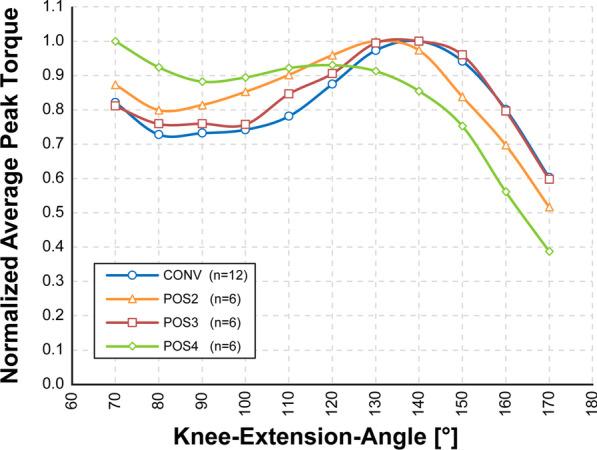
Relationship between torque and knee-extension-angle. The data displayed illustrates the relation between peak-torque and knee-extension angle (mean of each electrode configuration). Data was normalized to the individual maximal peak torque for each electrode configuration. For a better readability, SD is not shown in the diagram

The relation-ship between torque and knee-extension-angle was similar for most electrode configurations. Only POS4 showed a deviating shape and revealed its maximal peak-torque at the lowest knee-extension angle. For the other electrode configurations, the maximal peak torque was found on either 130° or 140° knee-extension-angle. After the 140° knee-extension a steep drop in peak-torque could be observed for all tested electrode configurations. Detailed traces of the average peak-torques for each knee-angle are illustrated in Fig. 3. Please note that CONV1 and CONV2 were treated together as they used the same electrode placement.

### Fatigue testing

Table 2 shows the normalized initial peak-torque values (average of first 10 contractions) and the corresponding stimulation amplitude as mean ± SD for each electrode configuration. Fatigue testing was performed at a targeted initial peak-torque of 40% of the MEC. This was achieved by adjusting the stimulation amplitude prior to the actual fatigue-test. There were no statistical differences in the initial peak-torques (p = 0.163) between any of the observed electrode configurations.

**Table 2:** Torque levels and stimulation intensity during fatigue testing

Electrode Configuration		CONV1	POS2	POS3	POS4	CONV2
Normalized Initial peak Torque	% of MEC	41.6 ± 2.7	39.1 ± 4.7	36.7 ± 2.9	39.4 ± 1.5	39.1 ± 2.7
Intensity at 40% MEC	mA	70.3 ± 10.1	59.7^*^ ± 3.3	50.2^*^ ± 4.0	53.2^*^ ± 5.3	66.8 ± 5.4

For the fatigue testing the stimulation amplitude was set to elicit contractions with a peak torque of 40% MEC. The table shows the normalized initial peak torque (average of the first 10 contractions) along with its corresponding stimulation amplitude. Data is represented as mean ± SD for each electrode configuration (n = 6). Fatigue measurements using the conventional electrode configuration were conducted as baseline measurements at the first (CONV1) and last (CONV2) session for each subject. Values marked with an asterisk (*) were statistically significant against CONV1 and CONV2.

All alternative electrode configurations (POS2, POS3 and POS4) had significantly lower (p < 0.05) stimulation amplitudes to reach 40% MEC, compared to conventional stimulation (CONV1 and CONV2). Stimulation intensities of POS2 were significantly higher compared to POS3 (p = 0.014) and POS4 (p = 0.014), but there was no statistical difference between POS3 and POS4 (p = 0.221).

**Fig. 4:**
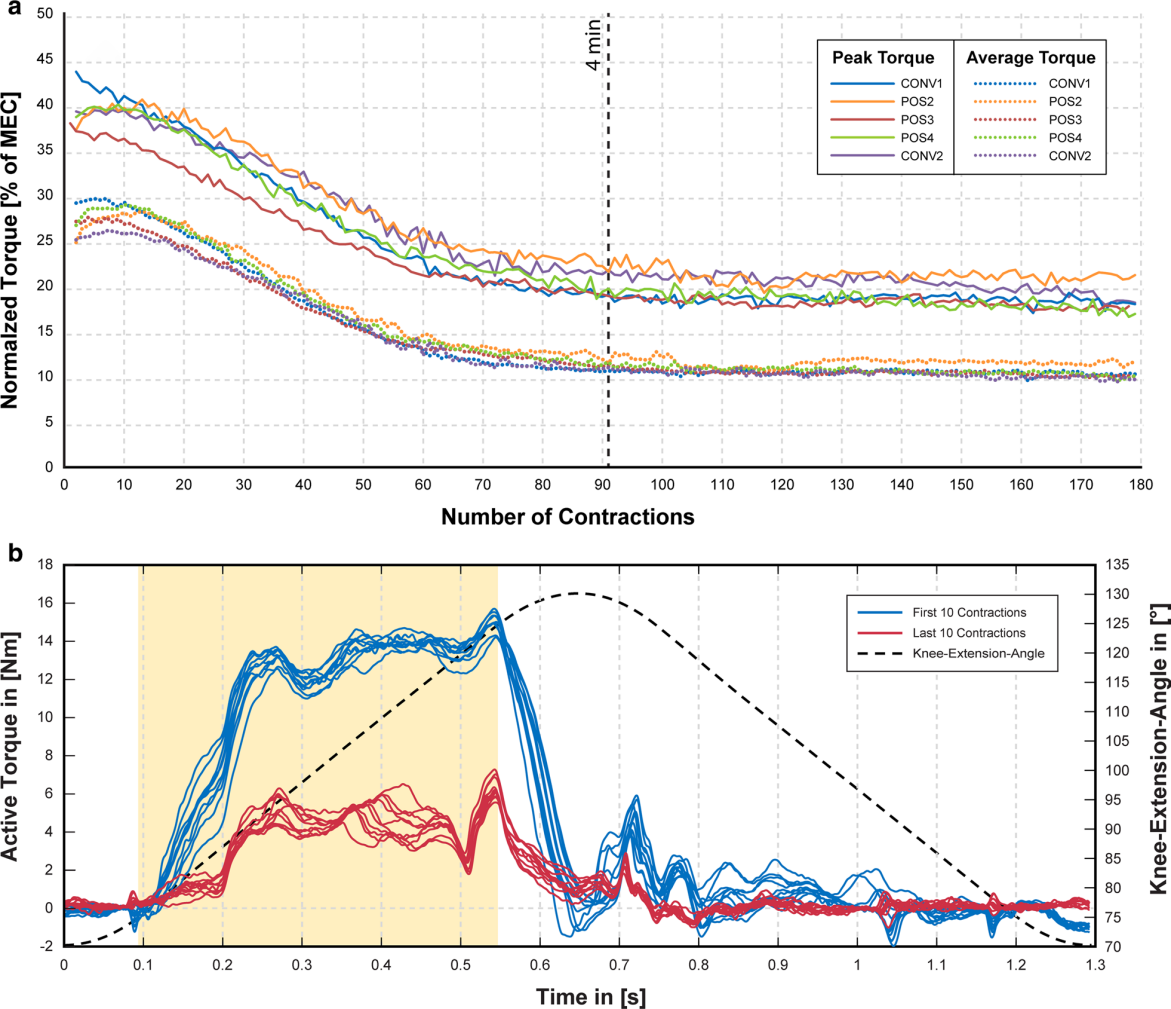
Fatigue testing. **a** Comparison of the fatigue development over 180 contractions using different electrode configurations. The means of all normalized peak-torque (solid traces) and normalized average-torque (doted traces) are presented for each electrode configuration (n ≥ 5). For a better readability, SD is not shown in the diagram. **b** Representative example of active torque traces throughout the extension/flexion cycle. The first 10 contractions (blue traces), in contrast to the last 10 contractions (red traces) are depicted along with the knee-extension-angle (black dashed trace). Stimulation was active during extension from 75° to 125° knee-extension angle and is highlighted in light orange

The development of fatigue over 180 contractions is illustrated in Fig. 4a. The figure shows the mean normalized peak and average torque for each of the investigated electrode configurations. Please note that only data-points with n ≥ 5 samples per electrode configuration (some individual contractions were manually excluded after visual inspection of the passive torque traces) are displayed in the figure. The fatiguing dynamics was relatively similar for all electrode configurations, i.e. the main loss of torque occurs within the first 90 contractions (~ 4 min of testing) while the torque remains almost constant afterwards. Although POS2 showed the highest peak (~ + 3% MEC) and average (~ + 1.5% MEC) values for τ_final_, there was no statistically significant difference against any of the other electrode configurations. Figure 4b shows a representative example of the first and last 10 active torque traces recorded during a complete extension-flexion cycle.

**Fig. 5:**
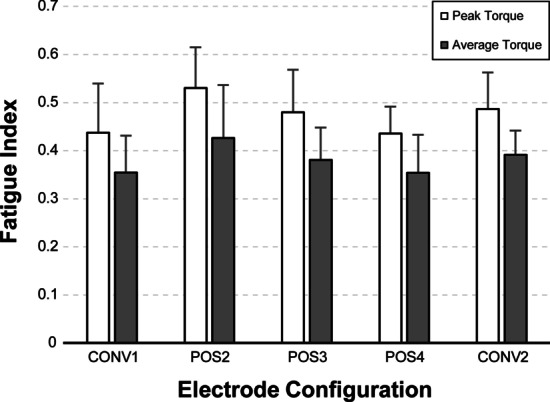
Fatigue indices of the tested electrode configurations. Fatigue indices were determined for peak and average torque of the last 10 contractions in relation to the first 10 contractions. Values are illustrated as mean ± SD. There were no statistically significant differences for any of the tested electrode configurations

The fatigue indices were calculated as quotients of τ_final_ divided by τ_init_ separately for peak and average torque (see Fig. 5). Assessing the fatigue index on basis of the peak-torque, reveals the following results for the individual electrode configurations: CONV: FI = 0.44 ± 0.10, POS2: FI = 0.53 ± 0.08, POS3: FI = 0.48 ± 0.09, POS4: FI = 0.44 ± 0.06 and CONV2: FI = 0.49 ± 0.08. Similar fatigue indices were obtained for average-torques: CONV: FI = 0.35 ± 0.11, POS2: FI = 0.43 ± 0.07, POS3: FI = 0.38 ± 0.08, POS4: FI = 0.35 ± 0.05 and CONV2: FI = 0.39 ± 0.08. Although, POS2 showed the highest fatigue indices, there were no statistically significant differences observed for any of the tested electrode configurations (FI evaluated on peak torques: p = 0.242, FI evaluated on average torques: p = 0.504).

## Discussion

The aim of our study was to investigate the influence of different electrode configurations and distributed stimulation on the fatigue resistance of paralysed quadriceps muscles during a dynamic knee-extension task. In literature, the use of distributed stimulation for artificial activation of the quadriceps has been shown beneficial to counteract muscular fatigue [6–14] during functional electrical stimulation. However, although the physiological principles are known since 1969 [5] and successfully demonstrated in the population with SCI more than 10 years ago [13], there is still no clinical implementation taking advantage of this technology.

One ideal application can be found in FES-assisted cycling. After conducting a targeted muscle training with FES, some patients with SCI are able to generate forces strong enough to allow for over-ground cycling, but their muscles are prone to fast fatigue within a few minutes. Here is where fatigue reducing techniques could make a major difference. Nevertheless, distributed stimulation is barely used outside of an experimental setting. In fact, even at the Cybathlon 2016 (a techno-sportive event with the intention to present state-of-the-art solutions for daily-life problems of the handicapped population) none of the participating teams was using distributed stimulation at the FES-cycling race [3].

A potential explanation could be that experimental results might not fully reflect the needs of a functional application. Many results are based on findings during isometric contractions [8, 12, 13]. Such measurements hold the advantage of a clean and reproducible measurement setup, but a direct translation of these findings into the regime of dynamic movements might be difficult, due to additional degrees of variation (e.g. changes of the electrode position relative to the nerves-fibres). As one of the main objectives of electrical stimulation lies in the restoration of functional movement, we believe that it is important to assess fatigue during dynamic movements as demonstrated before in [6, 9–11].

An important aspect of our study was to standardize the fatigue-assessment by conducting testing at a specified torque level which is related to the maximal torque production capability (maximal evoked contraction) of the muscle. While some studies described certain criteria for selecting initial values for fatigue testing, only Bergquist et al. [6] and Downey et al. [8] correlated their results with a measurement of maximal torque (maximum voluntary contraction of able bodies subjects, MVC). Bergquist et al. aimed to match the initial torque for each individual participant, but they did not match the torque across the different subjects (average initial torque 25% MVC, range 5—40% MVC). Downey et al. reports 10% MVC as initial torque for results obtained in able bodied subjects. For studies performed in SCI individuals no maximal torque or power values were reported.

Generally it is noticeable that dynamic fatigue-testing in subjects with SCI was performed at rather low values. For the individually tested legs, Laubacher et al. [9] reported initial average power-values in the range of 0.8 to 3.3 W, which are noticeably lower than what has been used in our study (range 11.2—23.2 W). This might be explained, by the fact that the authors recruited untrained subjects to participate in their study. We believe that it is essential, that participants have participated in a FES-based strengthening program for at least 4 months, to allow for greater forces and to increase reproducibility of the measurements. However, uttermost care needs to be taken in terms of patient safety during recruitment (e.g. bone status) and testing, as higher forces also increase the risk of a bone fracture [17].

Our objective to match the initial peak torque was achieved for all electrode configurations. Only POS3 had a slightly lower initial peak-torque compared to the target-value of 40% MEC. As the adjustment of the stimulation amplitude was conducted within only few dynamic contractions to avoid pre-fatiguing of the muscles, some variations in the initial peak-torque are to be expected. Nevertheless, we are confident that testing at an initial peak torque of 36.7% MEC (POS3) only had a minor impact on the fatigue-index.

Observing the required stimulation amplitudes to obtain 40% MEC reveals significantly lower stimulation amplitudes for all tested electrode configurations (POS2, POS3, POS4) compared to CONV. It is important to notice that this finding is not claiming a higher efficiency in stimulation. Instead of a single stimulation-channel (40 Hz) in CONV, POS2 is using two channels while POS3 and POS4 are using three channels to control the different parts of quadriceps muscles. The higher number of stimulating pulses therefore can be considered to be responsible for the lower stimulation amplitudes required to obtain the desired level of torque. Assessing stimulation efficiency was not the subject of this work.

The results of our study were not able to verify significant differences in the fatigue-resistance for any of the tested electrode-configurations compared to conventional stimulation. This finding was rather surprising as literature suggested clear benefits in the use of distributed stimulation. In fact, we had great expectations for POS4 as it was intended to apply the idea of distributed stimulation to 3 separate muscles of the quadriceps. One reason might be that many studies were performed in able-bodied subjects [6–8, 10, 11, 14], in which case the results need to be interpreted with caution. For an able-bodied person under electrical stimulation it can be challenging to fully relax. Unintended voluntary contractions, caused by unpleasant sensations as a side-effect of the electrical stimulation (e.g. pain), might influence the results. Concentrating on patients with SCI, Laubacher et al. [9] presented the power-output of 4 participants over the course of 160 contractions. They concluded higher fatigue-resistance using distributed stimulation based on higher overall power (mean over all contractions) and higher end power (mean of last 20 contractions). Although higher overall and final power-outputs are favourable parameters for a functional application, they do not directly reflect an appropriate measure of fatigue-resistance on their own. In fact, these particular parameters are more likely to be influenced by the more concentrated electrical field of the smaller electrodes, used during distributed stimulation. The authors also reported fatigue-indices individually for each leg, but did not provide statistical testing. Meta-analysis of their data (recomputed average based on Table 2 of [9], excluding right leg of P1 due to lack of power-output during conventional stimulation) revealed average fatigue indices of 0.72 ± 0.20 for distributed stimulation and 0.67 ± 0.28 for conventional stimulation. Although the FI shows a slightly better fatigue-resistance for distributed stimulation, it fails statistical significance (p = 0.257)—which is in line with our results.

Another explanation for our deviating findings could be that our levels of initial torque required higher stimulation amplitudes. It is noticeable that many studies [8–11] used current-pulses of 40 mA and phasewidths < 220 µs, while we delivered about 2–3 times the charge per pulse during our fatigue experiments. As all electrodes for distributed stimulation are positioned in close proximity of a motor-point, it is reasonable to assume that higher stimulation amplitudes cause a certain degree of spill-over and recruit additional motor-units indented to be stimulated by a neighbouring electrode. Due to the loss of intra-muscular selectivity, individual muscle-parts will thus successively receive a higher stimulation frequency when increasing the stimulation amplitude. This spill-over is not only limited to a particular motor-point and thus can also affect a neighbouring group of distributed stimulation of a different muscle. Therefore we hypothesize that the beneficial effects of distributed stimulation, regarding fatigue-resistance are less pronounced at higher stimulation amplitudes. This is of particular interest because of two reasons: (a) any practical application requires particular levels of force which only can be achieved at appropriate stimulation intensities; (b) a general approach for counteracting fatigue is to recruit new motor-units by increasing the stimulation intensity up to a predefined maximum. Although our results would be in favour of supporting the proposed hypothesis, further testing would be required for consolidation.

Stepping aside from surface stimulation – the technique of distributed stimulation might still be a powerful method to increase fatigue resistance when using a multi-contact cuff electrode implanted close to the nerve`s entry point of the targeted muscle. A localised electrical field combined with dedicated amplitude control for each electrode, might be a promising technique to counteract fatigue. Other methods like frequency modulation [18, 19], take advantage of the catch-like-property of a muscle [20], but still need validation for SCI-subjects within a practical measurement setup like [6, 7, 11] or our study.

Rapid onset of muscular fatigue still remains a major issue in the practical application of FES. Although methods for improved fatigue-resistance generally support their claims with statistically significant results, it is important to acknowledge that the overall fatigue-resistance during FES is still very low, compared to voluntary contractions. In the context of FES-cycling one can expect 500 contractions during a short duration 10 min cycling (at 50 RPM). In studies with SCI subjects, distinct levels of fatigue were already achieved within 30 [8] to 160 [9] contractions during a dynamic movement task—regardless of the stimulation technique used. While these low contraction numbers are certainly appropriate to reveal statistical differences, they also highlight the need for a better muscle control when using FES. In order to demonstrate a meaningful impact, it is necessary to validate potential improvements in a more practical setting, i.e. at higher forces and with a higher number of contractions. Improved motor-control might be achievable combining spatial (controlling separate pools of motor-units within a single muscle) and temporal (adjusting stimulation frequency for each pool) approaches, but more research is urgently needed to acquire a better understanding of the underlying principles and their application.

A secondary objective of the recent study was also to investigate the influence of different electrode configurations on the torque production for different knee-angles. Besides POS1 and POS4, our results show only minor differences between the different electrode configurations, confirming their stability with regard to movement-induced changes in the relative position between electrode and nerve.

POS1 was excluded from analysis, as we were unable to obtain reliable fused contractions with this electrode configuration. We mostly observed unstable contractions with high levels of variation between the different contractions. Although we kept the same activation order as described in [6, 12], our setup did not allow us to select a specific starting electrode (i.e. the first pulse getting delivered to the electrode 1), which we consider causing the instable behaviour. Nevertheless, we assume that POS1 still might be a promising electrode-configuration—if working. As it is targeting different motor points of different muscles, it allows for a clear separation of individual motor-units, which could have beneficial effects on fatigue resistance.

POS4 interestingly showed a deviating behaviour as it was reaching its highest torques at the lowest knee-extension-angle (70°). POS4 was positioned on the same pre-determined motor-points as POS3, but covered a slightly smaller electrode area. The position of the electrodes relative to the stimulated nerve is changing for different knee-extension-angles, which could explain the different torque production of POS4. All other configurations reached maximal-torque production at a knee-extension-angle of 130° or 140°, which matches the maximal knee-flexion-angles (range 35°–45°) reported during running [21], where it is important to effectively produce high forces. High torque (~ 85% of maximal torque) values were also observed at low knee-extension-angles. We assume that this might reflect the biomechanical properties of the individual muscles of the quadriceps, as it is acting over a wide knee-extension-angle. The quadriceps also has a significant contribution during movements at a very low knee-extension-angle, e.g. deep squatting exercises [22, 23].

In our study, physical limitations of the dynamometer did not allow for measurements at low knee-extension-angle in all of our subjects. Therefore we selected 70° knee-extension-angle as the lower limit. Although we assume that an additional second peak could be expected at lower knee-extension angles, further measurements would be required for verification – also additionally including different hip-angles. Information about the relationship between knee-extension angle and generated torque could be of great interest for mechanical engineers in order to design more efficient systems to harvest and use the generated torque in their applications (e.g. FES-cycling). Additionally it allows to verify the stability of a particular stimulation technique over the targeted range of motion, thus should be reported.

## Conclusions

One of the main problems of electrical stimulation in a practical functional application is the rapid onset of muscular fatigue compared to voluntary contractions. Although literature presents different techniques, such as distributed stimulation (distributing stimulation pulses across different portions of a muscle) to improve fatigue-resistance, there is still no translation of the proposed benefits into clinical rehabilitation or a practical application. Besides agreeing beneficial results for distributed stimulation, the great heterogeneity in methodology across different authors and studies, urges the need for a standardized method to better reflect the requirements during a practical application. Our study presents the development of fatigue in 6 paralysed legs through the course of 180 contractions, using different electrode configurations and distributed stimulation. To allow for a standardized comparison between the individual legs, fatigue-testing has been performed at 40% of the torque obtained during maximal evoked contraction (MEC).

None of the tested electrode-configurations showed a significant difference in the fatigue-index compared to conventional stimulation. As our fatigue-tests were performed at noticeably higher stimulation intensities (and thus at a higher torque, i.e. 40% MEC) than in literature, we hypothesise that the positive effects of distributed stimulation become less pronounced at higher stimulation amplitudes, due to decreasing selectivity of the individual surface electrodes. Most results demonstrating improved fatigue-resistance were obtained either under isometric conditions or in able-bodied subjects, which might not represent an appropriate model to reflect the demands of functional movement tasks (e.g. FES-cycling, FES-rowing…) in the population with SCI. Our study is the second study assessing FES induced fatigue in complete SCI subjects during a dynamic movement task. More research is urgently required to improve motor-control with the aim to delay the onset of muscular fatigue. Fatigue-resistance of novel stimulation techniques, should be evaluated by using a standardized method during a dynamic movement task. We suggest to perform fatigue testing at higher forces (e.g. 40% MEC) in pre-trained SCI subjects, to better reflect the demands during a practical FES application.

In the perspective of gaining a deeper understanding required to improve motor-control, future studies could use our proposed setup to investigate the influence of new electrode-configurations or alternative stimulation strategies (e.g. frequency modulation).

## Supplementary Information

**Additional file 1.** Anthropometric data for each participant. Contains a table with supplementary information about patient-specific characteristics.

## Data Availability

All required data and material has been included in the manuscript. High quality copies of the figures are submitted in separate files enclosed to the manuscript.

## Abbreviations

CONV: Electrode configuration for conventional stimulation
F: Frequency
FES: Functional electrical stimulation
FI: Fatigue Index
GPIO: General Purpose Input/Output
MEC: Maximal evoked contraction
MVC: Maximal voluntary contraction
PhW: Phasewidth
POS1: Electrode configuration 1
POS2: Electrode configuration 2
POS3: Electrode configuration 3
POS4: Electrode configuration 4
SCI: Spinal cord injury
SD: Standard deviation
τ_init_: Average torque of first 10 contractions
τ_final_: Average torque of last 10 contractions
