# Supplementary material for: Standardizing fatigue-resistance testing during electrical stimulation of paralysed human quadriceps muscles, a practical approach
Source: J Neuroeng Rehabil. 2021 Jan 21;18:11. doi: 10.1186/s12984-021-00805-7 (PMC7818559; doi:10.1186/s12984-021-00805-7)
Supplement: Supplementary file 1 — Additional file 1. Anthropometric data for each participant. Contains a table with supplementary information about patient-specific characteristics. [file 12984_2021_805_MOESM1_ESM.docx]

# Additional file 1

| Subject | Side | Length of Thigh | Length of Shank | Circumference Thigh | Midpoint shank fixation | MEC  (Mean ± SD) |
| --- | --- | --- | --- | --- | --- | --- |
|  |  | cm | cm | cm | cm | Nm |
| P1 | L | 42.0 | 42.0 | 47.0 | 33.0 | 38.0 ± 3.1 |
|  | R | 42.0 | 42.0 | 47.0 | 33.0 | 30.9 ± 2.5 |
| P2 | L | 42.5 | 40.5 | 51.0 | 32.0 | 34.9 ± 4.4 |
|  | R | 43.5 | 40.5 | 51.0 | 32.0 | 33.6 ± 2.9 |
| P3 | L | 47.0 | 48.0 | 46.0 | 37.5 | 35.0 ± 2.3 |
|  | R | 46.0 | 48.0 | 46.0 | 37.5 | 30.7 ± 0.9 |

**Table S1**: **Anthropometric data for each participant.** The length of the thigh was measured from the femoral head to the rotational center of the knee (lateral condyle). The length of the shank was measured from the rotational center of the knee (lateral condyle) to the rotational center of the ankle (lateral malleolus). The circumference of the thigh was obtained 15 cm above the patella. The midpoint of the shank fixation was measured from the rotational axis of the knee. The last column represents the peak torque during maximal evoked contractions (MEC) across all sessions.
